# LmaPA2G4, a Homolog of Human Ebp1, Is an Essential Gene and Inhibits Cell Proliferation in *L. major*

**DOI:** 10.1371/journal.pntd.0002646

**Published:** 2014-01-09

**Authors:** Brianna Norris-Mullins, Kaitlin VanderKolk, Paola Vacchina, Michelle V. Joyce, Miguel A. Morales

**Affiliations:** 1 Eck Institute for Global Health. Department of Biological Sciences. University of Notre Dame, Notre Dame, Indiana, United States of America; 2 Mass Spectrometry and Proteomics Facility, University of Notre Dame, Notre Dame, Indiana, United States of America; Louisiana State University, United States of America

## Abstract

We have identified LmaPA2G4, a homolog of the human proliferation-associated 2G4 protein (also termed Ebp1), in a phosphoproteomic screening. Multiple sequence alignment and cluster analysis revealed that LmaPA2G4 is a non-peptidase member of the M24 family of metallopeptidases. This pseudoenzyme is structurally related to methionine aminopeptidases. A null mutant system based on negative selection allowed us to demonstrate that LmaPA2G4 is an essential gene in *Leishmania major*. Over-expression of LmaPA2G4 did not alter cell morphology or the ability to differentiate into metacyclic and amastigote stages. Interestingly, the over-expression affected cell proliferation and virulence in mouse footpad analysis. LmaPA2G4 binds a synthetic double-stranded RNA polyriboinosinic polyribocytidylic acid [poly(I∶C)] as shown in an electrophoretic mobility shift assay (EMSA). Quantitative proteomics revealed that the over-expression of LmaPA2G4 led to accumulation of factors involved in translation initiation and elongation. Significantly, we found a strong reduction of *de novo* protein biosynthesis in transgenic parasites using a non-radioactive metabolic labeling assay. In conclusion, LmaPA2G4 is an essential gene and is potentially implicated in fundamental biological mechanisms, such as translation, making it an attractive target for therapeutic intervention.

## Author Summary

Leishmaniasis is a disease caused by protozoan parasites of the genus *Leishmania*. Its clinical manifestations are widespread, ranging from ulcerative skin lesions to life-threatening visceral infections. Approximately 1.5–2 million new cases of leishmaniasis are reported each year with an estimated 70,000 deaths. During the infectious cycle, *Leishmania* differentiates from the extracellular promastigote to the intracellular pathogenic amastigote form. Differentiation is triggered by environmental signals within the mammalian host, namely acidic pH and high temperature. Due to the absence of vaccination, chemotherapy, together with vector control, remains one of the most important elements in the control of leishmaniasis. Current anti-leishmanial drugs include pentavalent antimony, amphotericin B and miltefosine; most are toxic, expensive and risk becoming ineffective due to emerging resistance. Therefore, new drugs are urgently needed. LmaPA2G4 is a homolog of human proliferation-associated 2G4 protein (PA2G4, also termed Ebp1). We show that it is an essential gene in *L. major* and a gain-of-function approach allowed us to implicate LmaPA2G4 in translation and subsequent protein synthesis reduction, growth defects and virulence attenuation. This work highlights the essential role of LmaPA2G4 in the biology of the parasite and thus makes it an attractive target for drug development.

## Introduction

Protozoan parasites of the genus *Leishmania* are the causative agents of leishmaniasis, a disease that is characterized by a spectrum of clinical manifestations ranging from ulcerative skin lesions to fatal visceral infections [1]. Leishmaniasis is a poverty-related disease and is associated with malnutrition, displacement, poor housing, illiteracy, gender discrimination, weakness of the immune system and lack of resources [2]. Leishmaniasis is further compromised by the emergence of co-infection with human immunodeficiency virus (HIV) in endemic areas [3]. Globally, there are an estimated 1.5–2 million new cases of leishmaniasis and 70,000 deaths each year, and 350 million people are at risk of infection and disease [4]. Due to the absence of vaccination, chemotherapy, together with vector control, remains one of the most important elements in the control of leishmaniasis. Current anti-leishmanial drugs include pentavalent antimony, amphotericin B and miltefosine; most are toxic and expensive. To date, no successful vaccine exists and the few anti-leishmanial drugs mentioned risk becoming ineffective due to emerging resistances [5], [6]. Therefore, new drugs are urgently needed [7].

During the infectious cycle, *Leishmania* differentiates from the extracellular promastigote to the intracellular amastigote form. Flagellated promastigotes develop in the midgut of sandflies, and following infection in humans, differentiate to intracellular amastigotes that multiply inside the macrophage lysosome [8]. This differentiation is triggered by environmental signals, mainly acidic pH and high temperature in the mammalian host [9].

Signal transduction pathways often relay these environmental stimuli through reversible phosphorylation, ultimately leading to changes in protein activity, interaction and expression profiles [10]. Mitogen-activated protein kinases (MAPKs) are conserved virtually across all eukaryotic organisms. To gain insight into the MAPK pathway in *Leishmania* we performed comparative phosphoproteomics of MPK7 [11] and WT parasites with the objective of characterizing putative substrates of this kinase. As part of the screening we identified LmaPA2G4, a homolog of human proliferation-associated 2G4 (PA2G4, also termed Ebp1) [12]. PA2G4 proteins are highly conserved in eukaryotes and are involved in the regulation of cell growth and differentiation. The human member of this family, ErbB3 binding protein 1 (Ebp1), is ubiquitously expressed and localizes in both the nucleus and cytoplasm [13]. The protein binds structured RNAs and was suggested to be involved in linking ribosome biosynthesis and cell proliferation [14]. Here we show that LmaPA2G4 is an essential gene in *L. major*. The over-expression of LmaPA2G4 results in accumulation of intermediates of translation initiation and ultimately leads to growth and virulence defects.

## Materials and Methods

### Ethics statement

The University of Notre Dame is credited through the Animal Welfare Assurance (#A3093-01). All animal studies were conducted according to the Institutional Animal Care and Use Committee (IACUC) guidelines. The protocol for the infection of mice with *Leishmania* was approved by the University's IACUC (October 16, 2012, protocol #15-047).

### Parasite culture

*Leishmania major* strain Friedlin V1 (MHOM/JL/80/Friedlin) was cultured in M199 medium supplemented with 10% FBS at 26°C and pH 7.4 [15]. *L. donovani* strain 1S2D (MHOM/SD/62/1S-CL2D) was grown in M199 supplemented with 10% FBS and axenic amastigotes were differentiated as described previously [16]. For some experiments *L. major* metacyclic promastigotes were enriched by agglutination [17]. Briefly, cells were incubated for 30 min at RT with 50 µg/ml peanut agglutinin in M199 without serum, agglutinated parasites were removed by centrifugation and metacyclic parasites were recovered from the supernatant.

### Bioinformatics

*L. major* CAJ07101 (gi|68126048) was used as an initial query for PSI-BLAST and after four cycles results with significant E-values (<10e^−6^) were selected. Sequences corresponding to putative aminopeptidase proteins from the sequenced genomes of *H. sapiens*, *L. major*, *L. infantum*, *L. braziliensis*, *L. donovani*, *T. brucei*, *T. vivax*, T. *cruzi* and *T. congolense* were retrieved using TriTrypDB and UniProt databases; (http://tritrypdb.org/tritrypdb/) and (www.uniprot.org). Sequences were aligned with Clustal X (v 2.0). Alignments were converted to MEGA compatible files and fed into the MEGA5.2 software package. A Neighbor-Joining tree was computed with 500 bootstrap replicates.

### Molecular constructs and sequencing

In order to generate null mutants, a 901 bp region in the 5′ untranslated region (UTR) upstream of LmaPA2G4 was amplified with the primers 5′-ACCGGTACCCAATCATGGCCCACCGAAGG- 3′ (*KpnI*) and 5′-CGCCCCGGG / CTCGAGTTTTTTTGGGTGGGTGGC-3′ (*SmaI* or *XhoI*). A 903 bp fragment in the 3′UTR was amplified with primers 5′-ACCCTCGAG / GGATCCACGGCCGTGGCATCCGTG-3′ (*XhoI* or *BamHI*) and 5′-CGCGGTACCCACGATGGGCAGAACGCC- 3′ (*KpnI*). Reactions were performed in a total volume of 50 µl containing LongAmp high fidelity *Taq*- DNA polymerase (New England Biolabs) following the manufacturer's recommendation. Products were cloned into pGEM-T and pGEM-T Easy vectors (Promega) to create pGEM-T-5′UTR-3′UTR. A 2.8 kb *SmaI-XhoI* fragment from pX63HYG containing the hygromycin B (HYG B) gene and a 2.5 kb *XhoI-BamHI* fragment from PX63PAC including the puromycin (PAC) were ligated between 5′UTR and 3′UTR to generate the two targeting constructs. Constructs were linearized with *KpnI* and dephosphorylated. The LmaPA2G4 homolog (CAJ07101) was PCR amplified from genomic DNA of *L. major* FVI using the primers 5′-ACCAGATCTATGTCAAAGAACGCTGAC- 3′(*BglII*) and 5-GCGAGATCTCTACTTCGCGCGCTTCTT- 3′(*Bgl*II). Purified PCR products were cloned into pGEM-T EasyVector (Promega). N-terminal GFP-PA2G4 fusion and pXNG-PA2G4 were obtained by inserting the 1.1 kb *Bgl*II fragment from pGEM-T into the respective site of pXG-GFP+2 [18] and pXNG [19]. F1, R1 primer pair 5′- CATCAATATTTCATGCGC-3′ and 5′-CGTGTCCTCCTCTTCTTC- 3′; F2, R1 5′- GGTAGTGTCGCGTGTTGG-3′; F1, R2 5′-CTGCATCAGGTCGGAGACGC-3′ and F1, R3 5′-GGGGTCAGGGGCGTGGGTCAG-3′ were used to corroborate the absence of LmaPA2G4 ORF in the null mutant lines. LmaPA2G4 was PCR amplified and cloned into episomal vector pLEXSY (Jena Bioscience), and parasites were selected in 75 µg/mL hygromycin B (Sigma). Parasites transfected with the empty vector, pXG-GFP+2 and pLEXSY were used as mock controls. Null mutants and episomal transfectants were established by electroporation as previously described [20], [21].

### RT-PCR analysis

Total RNA was isolated from *L. major* and *L. donovani* WT and transgenic parasites with Trizol reagent (Life Technologies Inc., NY) using RNase-free plastic supplies. cDNA was amplified using M-MLV Reverse Transcriptase (Sigma) and oligo d(T)_15_ (Promega) following manufacturer's recommendations. PCR was carried out with LongAmp high fidelity *Taq*- DNA polymerase (New England Biolabs) using LmaPA2G4 specific primers 5′- CCACGTGGACGGCTACTGCGCCG-3′ and 5′- CTTCCTTTTCGAAGAGAATAGGG-3′ and GAPDH primers 5′- CGACGACGGCAAAGCAGAAG-3; and 5′- TCAGCGCCACACCGTTGAAG-3′. All RNA samples were treated with DNA-free (Ambion, Inc., TX) to remove contaminating genomic DNA. Each RT-PCR product was analyzed by gel electrophoresis using 1% agarose gels and band intensity analyzed with ImageQuant TL software (GE Healthcare).

### Confocal microscopy

Live *L. major* promastigotes over-expressing GFP-PA2G4 were immobilized on poly (L-lysine)-coated 35 mm glass bottom dishes (MatTek Corporation, USA) and counterstained with 1 µg/mL NucBlue Live Cell stain (Hoechst 33342) (Molecular Probes). Fluorescent imaging was performed using a spinning disk confocal Revolution (Andor Technology) and a 63× oil immersion objective. Image acquisition was done using AndorIQ software (Andor Technology) and images processed with ImageJ software (NIH, USA).

### Mouse infections

Virulence studies were performed as previously described [11]. Briefly, groups of five female BALB/c mice (Charles River) were injected in the footpad with 10^5^ metacyclic promastigotes from GFP-PA2G4, cured GFP-PA2G4 and mock control. Lesions were followed weekly by measuring the thickness of footpads with a Vernier caliper.

### Western blot analysis

Crude cell lysates were separated in 4–12% Bis-Tris NuPAGE gels (Life), and electro-blotted onto PVDF membranes (Pierce). Proteins were revealed using the following primary antibodies: mouse monoclonals anti-A2 (Abcam) and anti-GFP-HRP (Miltenyi Biotec), mouse monoclonal anti-tubulin (Sigma), and anti-rabbit or anti-mouse HRP-conjugated secondary antibodies (Pierce).

### In vitro kinase assay

20 µL of immuno-complexes (GFPK7; transgenic parasites over-expressing an active MPK7) were incubated in a Thermomixer R (Eppendorff) for 30 min at 30°C and 1000 rpm in a 50 µL reaction mixture (Millipore) containing 5 µg myelin basic protein (MBP) substrate (positive control) or recombinant substrate and 10 µCi [γ-^32^P] ATP (3000 Ci/mmol). Reactions were terminated by heating the samples for 10 min at 98°C in NuPAGE sample buffer and reducing agent (Life). 30 µL of the reaction were separated by SDS-PAGE. The gel was Coomassie-stained, fixed, dried and analyzed by autoradiography.

### Sample preparation and labeling for 2D- DIGE

Protein extracts from logarithmic *L. major* WT, GFP-PA2G4 and stationary GFPK7 promastigotes were differentially labelled with the spectrally resolvable Cy3 and Cy5 as previously described [21]. A pool of extracts was labelled with Cy2 for normalization purposes, following the manufacturer's recommendations (GE Healthcare). Phosphoproteins were enriched with affinity IMAC columns (Qiagen) as previously described [22]. Following labelling, proteins were precipitated using a 2-D Clean-Up kit (GE Healthcare), allowing for quantitative precipitation and removal of interfering substances, such as detergents, salts, lipids, phenolics, and nucleic acids.

### Electrophoretic Mobility Shift Assays

Synthetic double stranded RNA Poly (I∶C) (Sigma) was labeled with Label IT Cy5 labeling kit (Mirus). Briefly, 5 µg of RNA was incubated at a 1∶1 (v∶w) ratio of Label IT Cy5 reagent to nucleic acid. 40 ng of labeled Poly(I∶C) was incubated with 20 ng GFP-PA2G4 fusion protein in binding buffer (50 mM Tris pH 7.4, 0.5 mM EDTA, and 150 mM NaCl) at room temperature for 45 min. GFP-PA2G4 was immunoprecipitated with anti-GFP magnetic beads as previously described [20]. An aliquot of gel loading buffer (0.25% bromophenol blue, 0.25% xylene cyanol, 50% glycerol) was added to the reaction mixture and resolved on 10% non-denaturing polyacrylamide gels in 1× TBE. Gels were scanned on a Typhoon FLA 9500 Imager (GE Healthcare) using 633/670 nm for Cy5 filter and 489/508 nm for GFP filter.

### Isoelectric focusing (IEF) and two-dimensional gel electrophoresis (2D)

IEF of 100 or 120 µg of protein was carried out using an EttanIPGphor 3 System (GE Healthcare) at 20°C with 11 and 13 cm non-linear DryStrip (pH 4–7). Strips were passively rehydrated overnight at room temperature in rehydration solution (GE Healthcare) containing 0.5% IPG buffer 4–7 and the sample. The IEF maximum current setting was 50 µA/strip. The following conditions were programmed for IEF: 100 V gradient step for 5 h, 300 V gradient step for 5 h, 1000 V gradient step for 2 h, 6000 V gradient step for 8 h and 6000 V for 5 h (60550 Vh). Following IEF, strips were equilibrated in two different solutions for 15 min each (6 M urea, 75 mMTris/HCl pH 8.8, 29.3% glycerol, 4% SDS, 0.002% bromophenol blue) supplemented with 65 mM DTT and 13.5 mM iodoacetamide, respectively. The strips were transferred to SDS polyacrylamide gels and sealed with 0.5% agarose in 25 mMTris-base, 0.19 M glycine, 0.2% SDS, 0.01% bromophenol blue. Electrophoresis was carried out in an SE 600 Ruby cooled electrophoresis system (GE Healthcare) using 12.5% SDS-PAGE gels and two-step runs (1 W/gel for 15 min and 7 W/gel for 5 h).

### Staining procedures and image analysis

After electrophoresis, gels were scanned on a Typhoon FLA 9500 Imager (GE Healthcare) using 488/520 nm for Cy2, 532/580 nm for Cy3, 633/670 nm for Cy5 and 100 µm as pixel size. Gel images were normalized by adjusting PMT voltage to obtain appropriate pixel value without any saturation. Images were analyzed with Decyder v. 6.5 (GE Healthcare) and Delta2D v.4.3 software (Decodon). Gels were matched or warped and spots detected across all images. A 2-fold difference in abundance, with p-values<0.05, was considered significant for the expression profiles. Polyacrylamide gels were then fixed in 50% methanol and 7% acetic acid and stained using SYPRO Ruby total protein gel stain (Life). Spots of interest were manually excised from gels using a blue-light transilluminator (Life).

### Protein identification

The gel spots were subjected to reduction with 55 mM dithiothreitol (Sigma-Aldrich) in 25 mM ammonium bicarbonate (Fisher Scientific) at 56°C for 1 hour followed by alkylation with 100 mM iodoacetamide (Sigma-Aldrich) in 25 mM ammonium bicarbonate at room temperature in the dark for 45 min. The spots were washed with 25 mM ammonium bicarbonate for 10 min followed by two consecutive washes with 25 mM ammonium bicarbonate in 50/50 acetonitrile∶water for 5 min, each. The spots were placed in a vacuum concentrator to dry completely before the addition of 12.5 ng trypsin gold (Promega) to each gel spot. The spots were kept at 4°C for 30 min to swell and then were incubated at 37°C overnight. Following trypsin digestion, the supernatant was collected. Peptides were further extracted from the gel spots with two consecutive additions of 50% acetonitrile/45% water/5% formic acid to the spots followed by 30 min of vortexing. The two sets of extracts were combined with the supernatant from each gel spot and then vacuum concentrated to 10 µL. Each concentrated digest was desalted with a C18 Ziptip (EMD Millipore) according to the manufacturer instructions. The desalted digests were then dried down in a vacuum concentrator and reconstituted in 10 µL of 0.1% TFA in water. A 2 µL aliquot of each gel digest was injected onto a nanoAcquity UPLC (Waters Corporation) with a BEH300 C18 100 µm×100 mm column (Waters Corporation) with 1.7 µm particle size. A gradient of 0.1% formic acid in water (A) and 0.1% formic acid in acetonitrile (B) was performed starting with 2% B held for 6 min and then ramping to 40% B to 40 min and 90% B to 43 min. The column was washed with 90% B for 7 min and then re-equilibrated with 98% A: 2% B. The nanoAcquity was coupled to a LTQ Orbitrap Velos mass spectrometer (Thermo Corporation) for data dependent scans of the digested samples in which the top nine abundant ions in a scan were selected for CID fragmentation. The UPLC-MS/MS chromatograms and spectra were analyzed using Xcalibur software (Thermo), and the extracted data were searched against the *L. major* custom database via Mascot and/or Protein Pilot. Search criteria included a global modification of carbamidomethylation on the cysteines. Proteins identified had less than a 1% false discovery rate.

### Metabolic labeling

The metabolic labeling of *de novo* synthetized proteins was conducted using the non-radioactive assay Click-iT AHA kit and Click-iT Cell Reaction Buffer Kit (Life) following manufacturer's guidelines with minor modifications. Briefly, 2×10^8^ mid-log phase WT and GFP-PA2G4 *L. major* and *L. donovani* promastigotes as well as GFP-PA2G4 *L. donovani* amastigotes were initially incubated for 30 min at 27°C in 2 mL methionine-free RPMI medium supplemented with 10% FBS in order to deplete methionine reserves. Metabolic labeling was performed for 2 h at 27°C in presence of 50 µM azidohomoalanine (AHA). A culture of *L. major* treated for 2 h with 100 µg/mL cycloheximide, an inhibitor of protein biosynthesis, was included as a positive control. After labeling, cells were harvested and lysed and 50 µL of each sample was employed to perform the Click reaction with TAMRA. Proteins were precipitated, resolubilized in 1D gel electrophoresis sample loading buffer and heated for 10 min at 70°C and subsequently resolved in a precast polyacrylamide gel (NuPAGE Novex 4–12% Bis-Tris gels, Life). Gel was visualized in a Typhoon FLA 9500 (GE Healthcare) and analyzed with ImageQuant TL software (GE Healthcare). After imaging the gel with TAMRA-labeled samples, the gel was fixed and stained with SYPRO Ruby in order to assess total protein content. Statistical comparisons were made using non parametric Mann–Whitney U-test.

## Results

### Identification of LmaPA2G4

**Figure 1:**
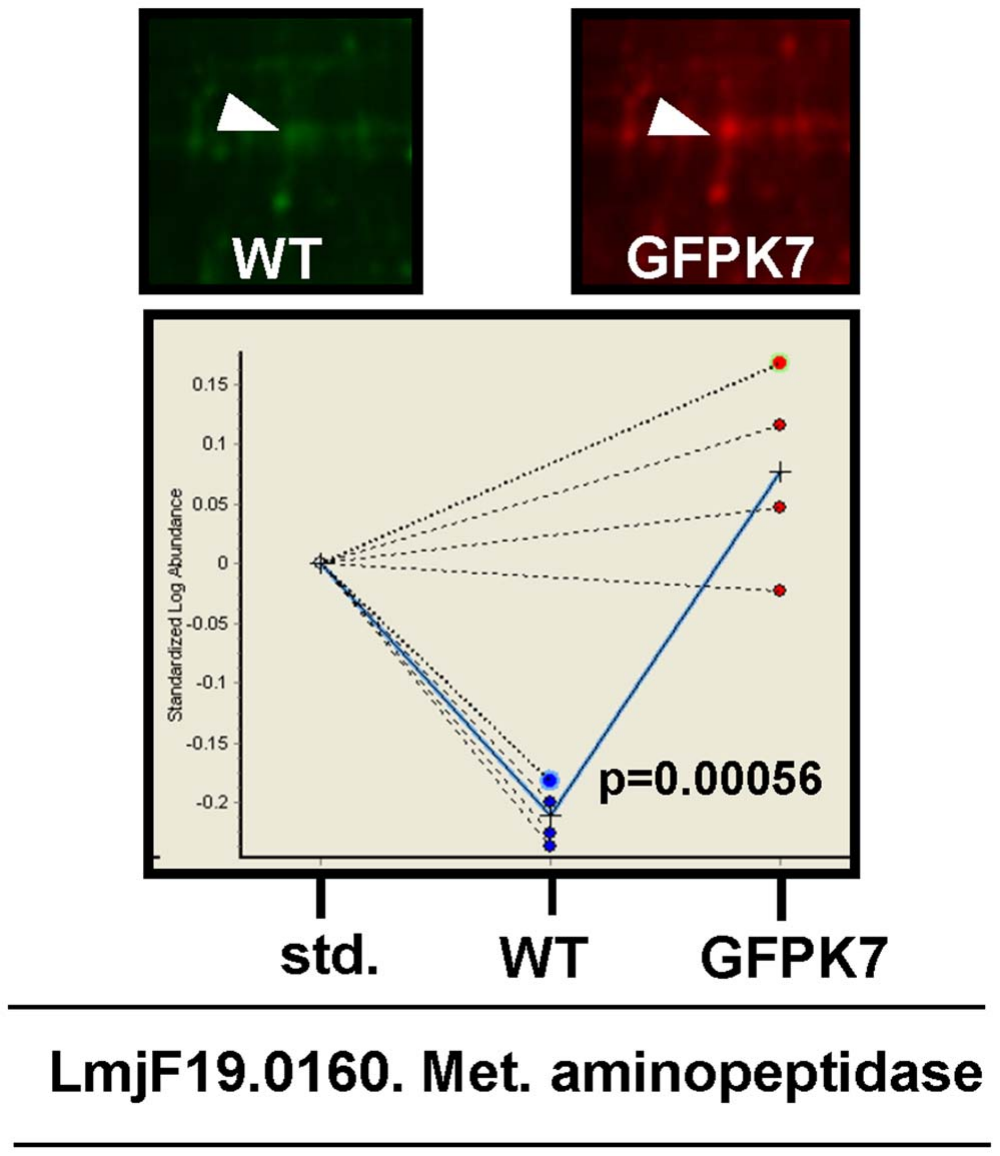
2D-DIGE quantitative phosphoproteomics analysis of GFPK7. An enlarged region of the 2D-DIGE gels showing Cy3-labeled WT stationary promastigotes and Cy5-labeled stationary GFPK7 promastigotes is presented. Spot ID 207 (white arrow) was over-represented in GFPK7. In the lower panel, a graphical representation of the BVA (Biological Variation Analysis) module of Decyder software (GE Healthcare) with statistics for spot ID 207 (2.97 fold and p = 0.00056). The spot was analyzed by mass spectrometry and identified as LmjF19.0160, a putative aminopeptidase.

We have previously shown that *L. major* MPK7 is implicated in parasite growth control, including the pathogenic amastigote stage [11]. The overexpression of an active MPK7 (GFPK7 transgenic parasites overexpressing MPK7) led to defects in cell cycle and, ultimately, attenuated virulence in a mouse model. In order to identify potential downstream targets of MPK7 we performed comparative 2D-DIGE of phosphoproteins isolated from four independent stationary cultures of *L. major* wild type (WT) and GFPK7 promastigotes. MPK7 shows increased activity at stationary phase [20]. Phosphoproteins were isolated by immobilized metal affinity chromatography (IMAC) and differentially labeled with CyDye fluors (GE Healthcare) as detailed in Material and Methods. Phosphoproteins were separated by 2DE on pH 4–7 IPG immobiline strips and SDS-PAGE. Images were analyzed with DeCyder 6.5 software (GE Healthcare). Up-regulation of spots more than 2-fold in GFPK7, with p-values<0.05 were considered significant. Spot ID 207 was over-represented in GFPK7 (2.97 fold change and p = 0.00056) and excised from the gel and analyzed by MS/MS (Fig. 1). LmjF19.0160 (Tritryp gene ID) is a putative aminopeptidase with a predicted MW of 43 kDa. Recombinant LmjF19.0160 was not phosphorylated *in vitro* by active GFPK7 using an in vitro kinase assay (Fig. S1). Phosphotransferase activity of recombinant GFPK7 was assessed by phosphorylation of MBP. Given the fact that LmjF19.0160 was enriched with an IMAC (phospho-specific) column, we are attempting to characterize the putative phosphorylation sites, with the objective of performing site-directed mutagenesis.

### Bioinformatics analysis of *L. major* LmjF19.0160

**Figure 2:**
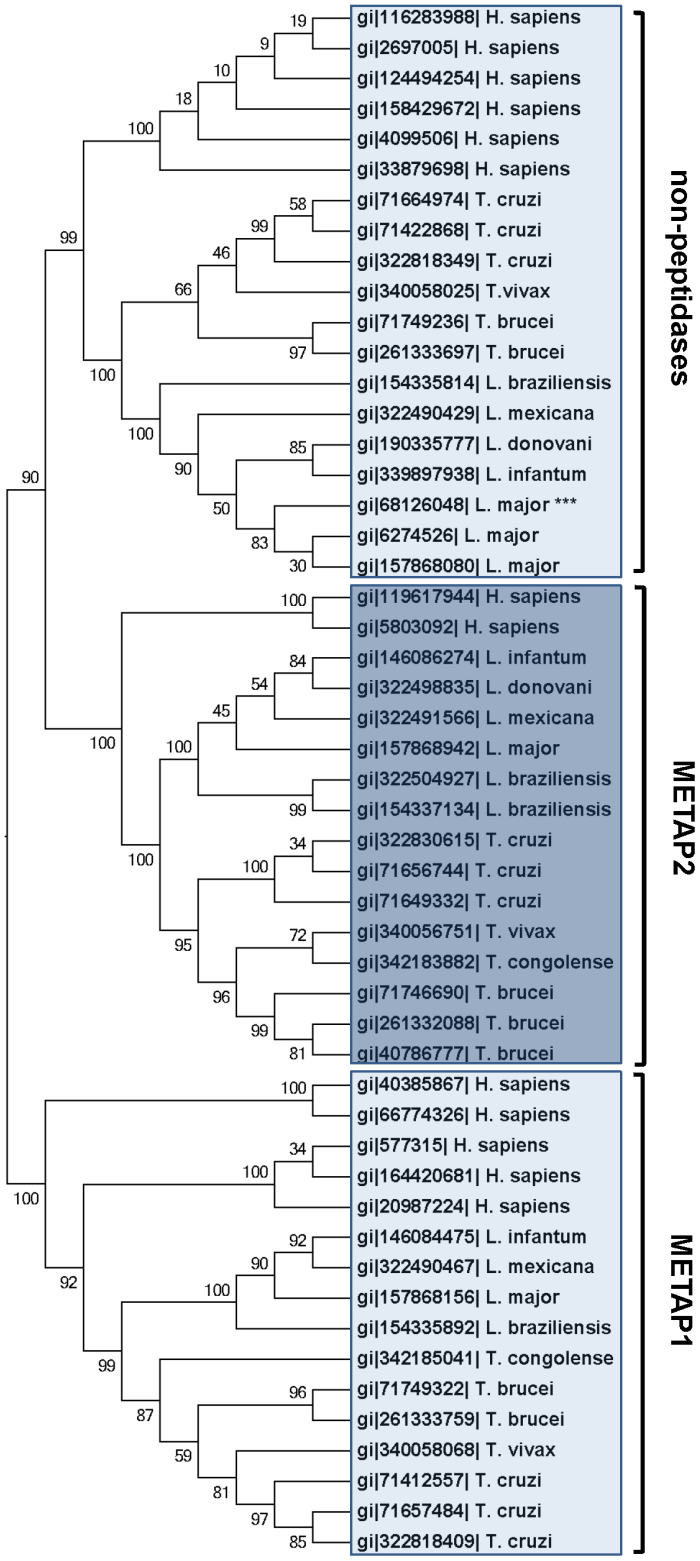
Bioinformatics analysis of *L. major* LmjF19.0160. Relationship between human and trypanosomatid members of the M24A subfamily of metallopeptidases was analyzed by multiple alignment and cluster analysis using Clustal X. This subfamily comprises homologs of methionyl aminopeptidases 1 and 2 (METAP1 and 2), and non-peptidases. Alignment was fed into MEGA5.2 software and a Neighbor-Joining tree was computed with 500 bootstrap replicates. Numbers on nodes indicate bootstrap support.

LmjF19.0160 belongs to the clan MG, family M24 of metallopeptidases according to the classification of MEROPS database [23]. Family M24 is further divided into subfamilies M24A and B. Typical members of subfamily M24A are methionyl aminopeptidases type I and II (METAP1 and 2). These peptidases are essential for the removal of the initiating methionine of many proteins [24]. We investigated the relationship between human and trypanosomatid members of the M24 family of metallopeptidases by multiple alignment and cluster analysis. LmjF19.0160 (protein ID gi|68126048) was used as an initial query for PSI-BLAST against the sequenced genomes of *H. sapiens*, *L. major*, *L. infantum*, *L. braziliensis*, *L. donovani*, *T. brucei*, *T. cruzi*, *T. vivax* and *T. congolense*. Homologs of human METAP1 and 2 are found in all trypanosomatids as shown by the clustering tree (Fig. 2). Bootstrap values support the existence of METAP1 and 2 subclasses among *Leishmania* and *Trypanosoma*. Interestingly, LmjF19.0160 clusters with homologs of the human proliferation-associated protein 2G4 (PA2G4) [25]. These are non-peptidase proteins which possess the “pita-bread” fold typical of methionyl aminopeptidases, however they lack metal cofactors and peptidase activity [26].

### RT-PCR analysis

Although the genome of *Leishmania* seems to be constitutively expressed [27], between 6% and 9% of the genes display significant expression profiles. Therefore, we analyzed the transcript levels of LmaPA2G4 by semi-quantitative RT-PCR. Total RNA was isolated from *L. major* logarithmic and metacyclic parasites. Peanut agglutination was used to enrich metacyclics in stationary cultures [28]. For *L. donovani* we used host-free amastigotes as previously described [16]. GAPDH was used as a housekeeping gene for semi-quantification purposes. As judged by the LmaPA2G4/GAPDH ratio, there are not significant differences in the expression levels across different life stages (Fig. S2).

### Loss-of-function studies in LmaPA2G4

**Figure 3:**
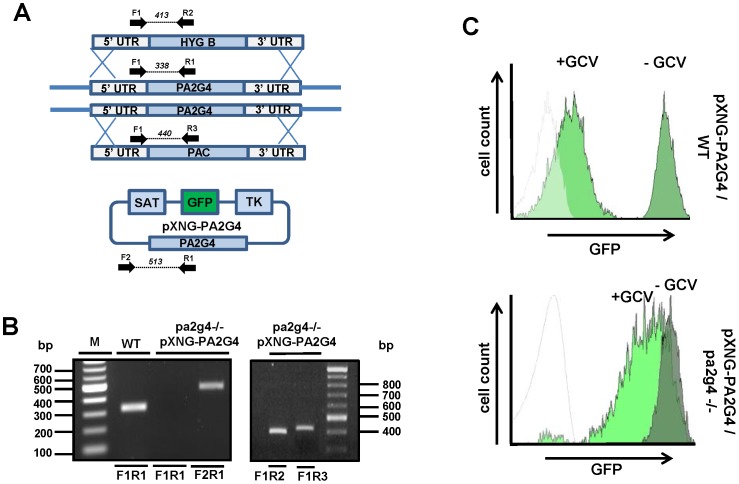
Establishment of L. major PA2G4 conditional null-mutant parasites. (A) Schematic representation of the null-mutant strategy. Two alleles of LmaPA2G4 were replaced by homologous recombination with hygromycin B (HYG B) and puromycin (PAC) resistance markers. Replacement was performed in the presence of an ectopic copy of PA2G4 (pXNG- PA2G4), carrying a nourseothricin (SAT) marker, a fluorescent protein (GFP) and a *Herpes simplex* virus thymidine kinase (TK). (B) PCR analysis of total DNA from wild-type (WT) parasites or one independent clone (PA2G4 −/− [pXNG-PA2G4]). F1,R1 primers (expected size 338 bp) confirm the presence of the endogenous copy of PA2G4, while F2,R1 primers (expected size 513 bp) confirm of the episomal PA2G4 ORF. F1, R2 primer pair (expected size 413 bp) and F1, R3 (expected size 440 bp) show the integration of hygromycin b and puromycin genes, respectively. Molecular weight marker (M) is shown. (C) (Upper panel). WT parasites carrying a copy of pXNG-PA2G4 were selected and grown in SAT and the GFP intensity was analyzed by flow cytometry (GCV−). After negative selection with the addition of 50 µg/mL GCV (GCV+) to the culture (3 passages) a reduction in GFP fluorescence is observed (lower panel). In contrast, conditional PA2G4 null-mutant parasites retained the ectopic copy of pXNG-PA2G4 as shown by the minimal reduction in GFP intensity. Histograms plots of one representative analysis are shown. Dotted lines correspond to fluorescence background levels of control parasites.

In order to gain insight into the putative function of PA2G4 in *Leishmania* we designed a loss-of-function strategy. *Leishmania* are diploid parasites and two rounds of targeted replacement with a drug-resistance marker are necessary. Unsuccessful attempts to replace the two PA2G4 alleles with resistance markers, to create a homozygous KO, suggested that LmaPA2G4 may be an essential gene. To demonstrate the essentiality of LmaPA2G4 we used a genetic method based on negative selection [19] to guard against the potential lethal phenotype. Both LmaPA2G4 alleles could be removed by homologous recombination in the presence of an episome expressing LmaPA2G4 (pXNG-PA2G4) (Fig. 3A). The loss of endogenous LmaPA2G4 in the null mutants was confirmed in two independent homozygous lines by PCR (Fig. 3B). As expected, only the episomal copy of LmaPA2G4 is present. The episome pXNG [19] carries a negative selectable thymidine kinase (TK), a fluorescent protein (GFP) and a resistance marker (SAT). TK renders the parasites susceptible to ganciclovir (GCV). PXNG-PA2G4/WT parasites were selected and grown in the presence of 250 µg/mL nourseothricin (SAT) and the GFP intensity was analyzed by flow cytometry. After negative selection with the addition to the culture of 50 µg/mL GCV during three passages, a dramatic shift in GFP fluorescence was observed (Fig. 3C, upper panel). However, after negative selection with GCV, mutant LmaPA2G4 parasites retained the ectopic copy of pXNG-PA2G4, as shown by the minimal reduction in GFP intensity (Fig. 3C, lower panel). These results suggest that LmaPA2G4 is an essential gene in *L. major*.

### Phenotypic characterization of GFP-PA2G4 parasites

**Figure 4:**
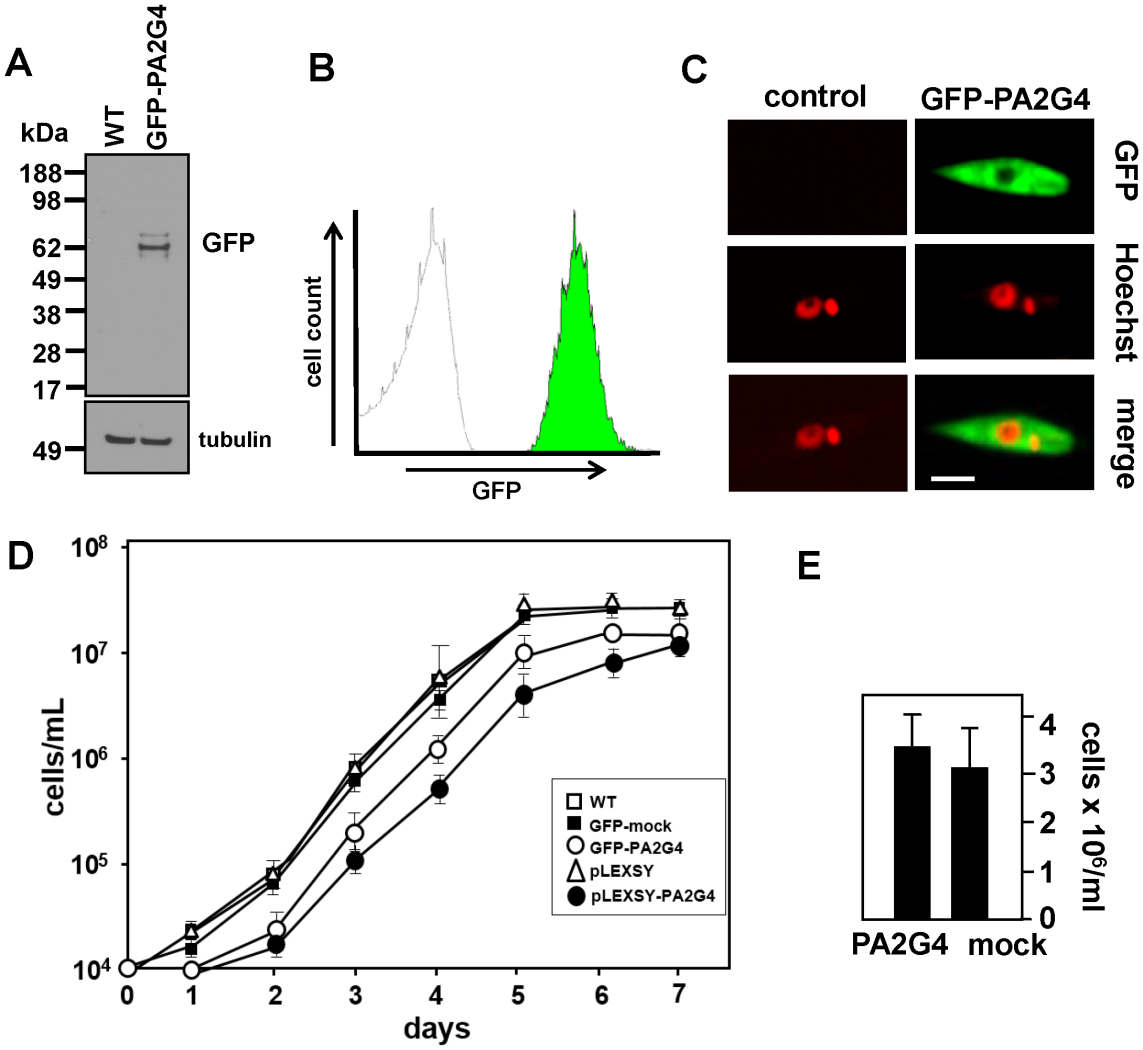
Gain-of-function strategy to study LmaPA2G4. (A) WT or parasites over-expressing an N-terminal GFP-PA2G4 fusion protein were lysed, resolved by SDS–PAGE, electroblotted on a PVDF membrane and analyzed by western blot using monoclonal anti-GFP antibody and anti-tubulin as a loading control. The molecular weight of standard proteins is indicated in kDa. (B) GFP intensity in GFP-PA2G4 transgenic promastigotes was analyzed in a Beckman Coulter FC-500 flow cytometer. 10,000 events were recorded. GFP mock control parasites are clearly distinguished from GFP-PA2G4. (C) Live control and transgenic promastigotes from logarithmic culture were analyzed using spinning disk confocal microscopy. Nuclei were counterstained with 1 µg/mL NucBlue Live Cell stain (Molecular Probes) (red). The bar corresponds to 7 µm. (D) Growth curve of *L. major* WT, GFP-mock, GFP-PA2G4, pLEXSY and pLEXSY-PA2G4 promastigotes was measured microscopically by counting cells in a Neubauer chamber. The average and standard deviation of triplicate determinations are shown. (E) The infective metacyclic stage in control and transgenic lines was analyzed. Parasites from five day stationary cultures were incubated with peanut agglutinin and numbers of non-agglutinating metacyclics were determined microscopically. Two independent experiments were performed and one representative triplicate experiment with standard deviation denoted by the bars is shown.

Since the essentiality of LmaP2G4 precluded further loss-of-function analysis, we followed a gain-of-function strategy to reveal the implication of LmaPA2G4 in the biology of *Leishmania*. We created parasites over-expressing an N-terminal GFP-PA2G4 fusion protein. LmaPA2G4 ORF was cloned into pXG-GFP2+ as previously described [20]. We confirmed the fusion by western blot analysis of *L. major* FVI wild-type (WT) and transgenic GFP-PA2G4 promastigotes using monoclonal anti-GFP and tubulin as a loading control (Fig. 4A). Fluorescence intensity of GFP-PA2G4 parasites was measured by flow cytometry (Fig. 4B). Live log-phase transgenic promastigotes were immobilized on poly(l)lysine-coated 35 mm glass bottom dishes and cells were analyzed using spinning disk confocal microscopy (Fig. 4C). Nuclei were counterstained with NucBlue Live Cell stain (Molecular Probes) (red). The ectopic expression of LmaPA2G4 is predominantly cytoplasmic and the overexpression had no effect on the morphology and viability of the parasites. Growth curves of WT, GFP-PA2G4 and GFP-mock control show that the overexpression of LmaPA2G4 results in a significant growth delay (Fig. 4D). LmaPA2G4 growth defect was reproduced by transgenic parasites expressing untagged protein (pLEXSY-PA2G4) and thus is independent from GFP expression. We also analyzed the infective metacyclic stage in control and transgenic lines. Similar numbers of metacyclic parasites were agglutinated in both lines (Fig. 4E), indicating that the over-expression of LmaPA2G4 does not affect metacyclogenesis.

### GFP-PA2G4 transgenic parasites are attenuated in virulence

**Figure 5:**
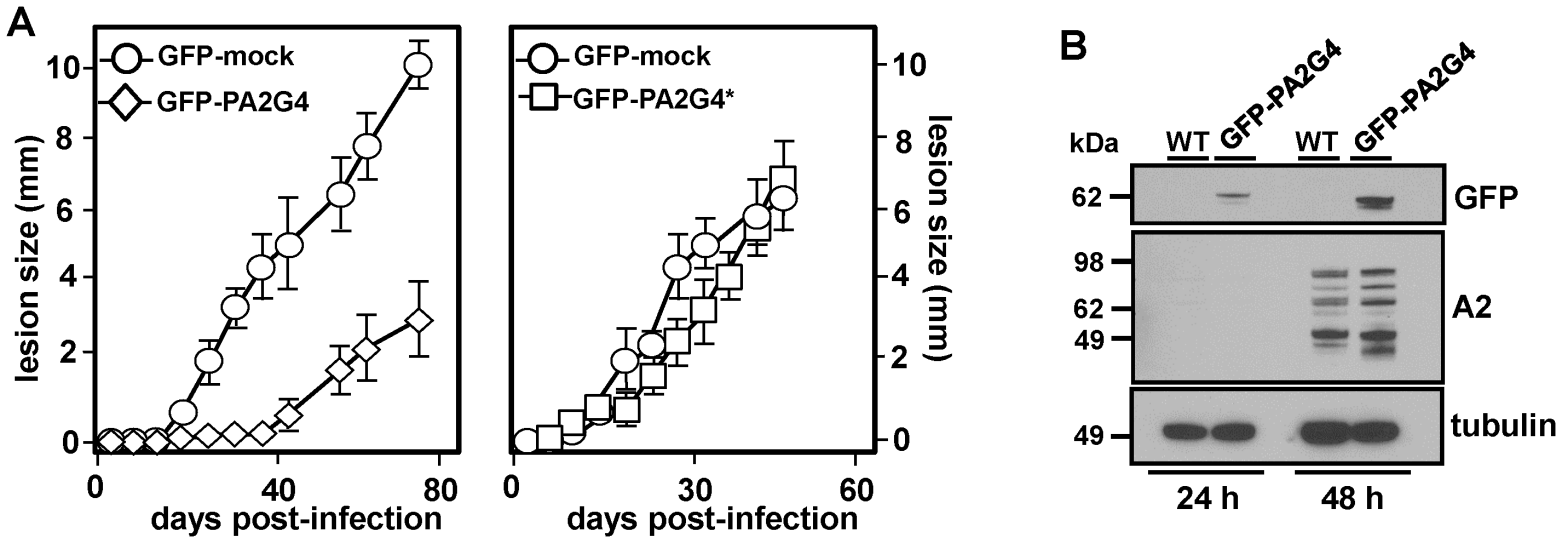
Attenuated virulence in GFP-PA2G4 transgenic parasites. (A) 10^5^ *L. major* metacyclic mock and transgenic parasites expressing GFP-PA2G4 and cured GFP-PA2G4 grown in the absence of G418 were inoculated into the footpad of female BALB/c mice. Lesion formation was followed by measuring the increase in footpad size with a Vernier caliper. Groups of five mice were analyzed and standard deviation is indicated by the bars. Two independent experiments were performed and one representative experiment is shown. (B) *L. donovani* transgenic (GFP-PA2G4) lines were established. 2×10^5^ promastigotes were inoculated in low pH medium and 37°C to trigger differentiation to axenic amastigotes. Cells were lysed 24 and 48 h after differentiation, and western blot performed with anti-GFP, amastigote-specific A2 antibody and anti-tubulin as a loading control. The molecular weight of standard proteins is indicated in kDa.

We investigated the effects of LmaPA2G4 overexpression on parasite virulence using an established experimental mouse infection [29]. Mock, GFP-PA2G4 and cured GFP-PA2G4* parasites grown in G418-free medium were normalized for virulence through one passage in BALB/c mice [30]. 10^5^ mock, GFP-PA2G4 and GFP-PA2G4* metacyclic parasites were inoculated into the hind footpad of groups of five female BALB/c mice. Lesion formation was followed by measuring the increase in footpad size with a Vernier caliper. Mock and GFP-PA2G4* parasites elicited a strong response *ca.* 30 days after inoculation and resulted in necrotic lesions (Fig. 5A). Interestingly GFP-PA2G4 are highly attenuated and lesions were only apparent after 40 days after inoculation. The cured line, grown in the absence of G418, elicited a response similar to GFP mock parasites, suggesting that the specific expression of LmaPA2G4 is responsible for the attenuated phenotype. At least two independent GFP-PA2G4 lines were used to rule out potential discrepancies due to clonal variations. To determine whether the overexpression of LmaPA2G4 affects the differentiation from pro- to amastigotes, we established *L. donovani* transgenic (GFP-PA2G4) lines that allow axenic amastigote differentiation. 2×10^5^ promastigotes were inoculated in low pH medium and 37°C to trigger differentiation [9]. We monitored the axenic amastigotes 24 and 48 h after differentiation (Fig. 5B). As judged by the expression of the amastigote-specific A2 protein family [31], transgenic parasites carrying GFP-PA2G4 are *bona fide* amastigotes at 48 h and no differences are observed when compared with WT. This result suggests that the virulence attenuation is potentially due to defects in cell proliferation.

### 2D-DIGE comparative proteomics analysis

**Figure 6:**
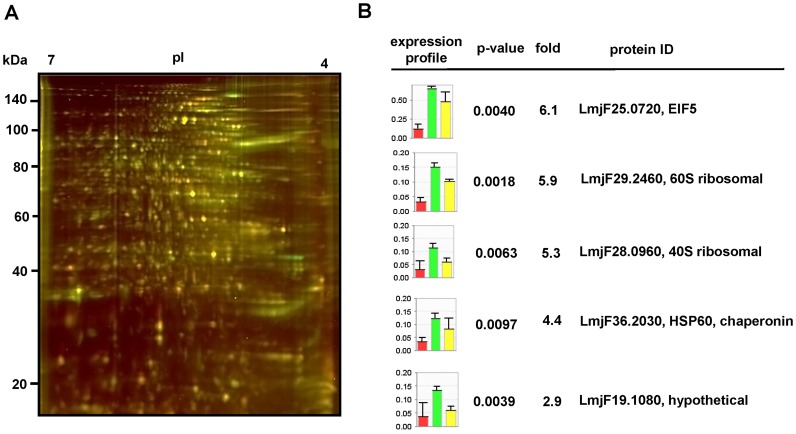
2D-DIGE analysis of the *L.major* mock and GFP-PA2G4 proteome. (A) Representative 2D-DIGE gel. Protein extracts from promastigotes of three independent biological experiments were differentially labeled with the CyDye fluors Cy3, Cy5 and Cy2, and separated by two-dimensional electrophoresis on 13 cm pH 4–7 IPG strips and 12.5% polyacrylamide gels. Gels were scanned on a Typhoon FLA 9500 (GE Healthcare) imager. A merged image of Cy5-labeled GFP-mock (red) and Cy3-labeled GFP-PA2G4 (green) is shown. The molecular weight of marker proteins and the pI range of the IEF gradient are indicated. (B) Differences in protein abundance were revealed by analyzing the gel images with Delta2D v4.3 software (Decodon). Histograms are a graphic representation of these significant differences. For normalization purposes, a Cy2-labeled internal standard was included, corresponding to a pool of protein from all extracts used in the analysis (yellow bars). Fold change and p-values of the spots selected are shown, as well as protein identification by mass spectrometry. Raw data of MS data and Mascot searches is presented in Table S1.

To gain a better insight into the function of LmaPA2G4, we quantitatively compared protein extracts from *L. major* GFP-PA2G4 and mock promastigotes of three independent biological repeats. Protein samples were differentially labelled with CyDye fluors (GE Healthcare) and separated by two-dimensional electrophoresis (2DE) on IPG strips and polyacrylamide gels as previously described [21]. A representative merged image of Cy5-labeled GFP-mock (red) and Cy3-labeled GFP-PA2G4 (green) is shown (Fig. 6A). Gels were scanned on a Typhoon FLA-9500 Imager and analyzed by Delta2D v 4.3 (Decodon) software package. Figure 6B shows a graphical representation of the expression profiles of mock (red) and GFP-PA2G4 (green) samples. Five spots with significant expression differences in GFP-PA2G4 (p-value<0.005) were selected. Gels were stained with the fluorescent stain SYPRO Ruby and the five spots of interest were excised and identified by MS/MS. Interestingly, the homologs of identified eukaryotic translation initiation factor 5 (LmjF25.0720, 6.1-fold change), 60S ribosomal protein (LmjF29.2460, 5.9-fold change) and 40S ribosomal protein (LmjF28.0960, 5.3-fold change) are implicated in translation initiation and elongation [32]. The chaperonin HSP60 (LmjF36.2030, 4.4-fold change) is involved in stress response and acts as a catalyst of folding proteins [33]. Raw data of protein identification and Mascot searches is presented in Table S1.

### Electrophoretic Mobility Shift Assays (EMSA)

**Figure 7:**
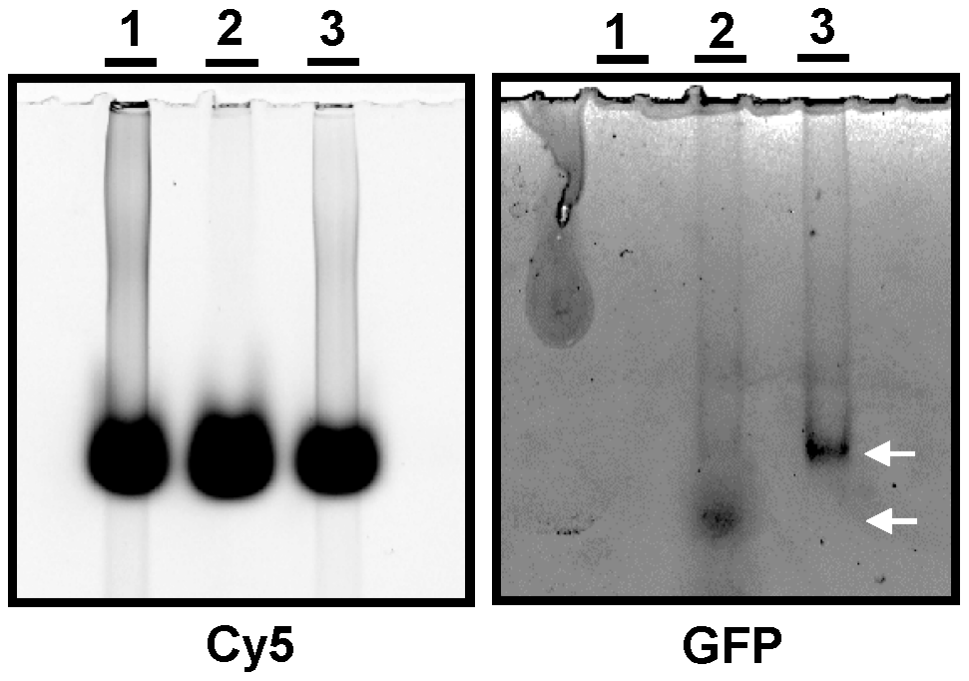
Electrophoretic Mobility Shift Assay. Poly (I∶C), a synthetic double stranded RNA was labeled with Cy5 and incubated with 20 ng GFP-PA2G4 fusion protein at room temperature for 45 min. The reaction was resolved in 10% non-denaturing polyacrylamide gels. Lane 1: 40 ng poly (I∶C); lane 2: 20 ng GFP-PA2G4; lane 3: 20 ng GFP-PA2G4 incubated with 40 ng poly (I∶C). Gel was scanned on a Typhoon scanner (GE) using 633/670 nm for Cy5 and 489/508 nm for GFP. Arrows indicate mobility shift of GFP-PA2G4.

dsRNA-binding domains characterize an expanding family of proteins involved in different cellular processes, ranging from RNA editing and processing to translational control. Human homolog Ebp1 interacts with double stranded RNA [34] and thus it was tempting to study whether GFP-PA2G4 is able to bind a synthetic double stranded RNA Poly (I∶C). RNA molecules (Sigma) were labeled with Cy5 in order to normalize and visualize the reaction. 40 ng labeled Poly(I∶C) was incubated with 20 ng GFP-PA2G4 protein in binding buffer at room temperature for 45 min and resolved on 10% non-denaturing polyacrylamide gels in 1× TBE. Gel was scanned in a Typhoon imager (GE) with Cy5 filters. Poly (I∶C) is visible in lanes 1 and 3 (Fig. 7, left panel). Cy5 filters produce a non-specific background signal with xylene cyanol, which is part of the loading buffer. Taking advantage of the GFP fusion, the gel was re-scanned with a GFP filter, and arrows indicate the apparent mobility shift of GFP-PA2G4 in the presence of Poly (I∶C) (Fig. 7, right panel).

### *De novo* protein synthesis in parasites over-expressing LmaPA2G4

**Figure 8:**
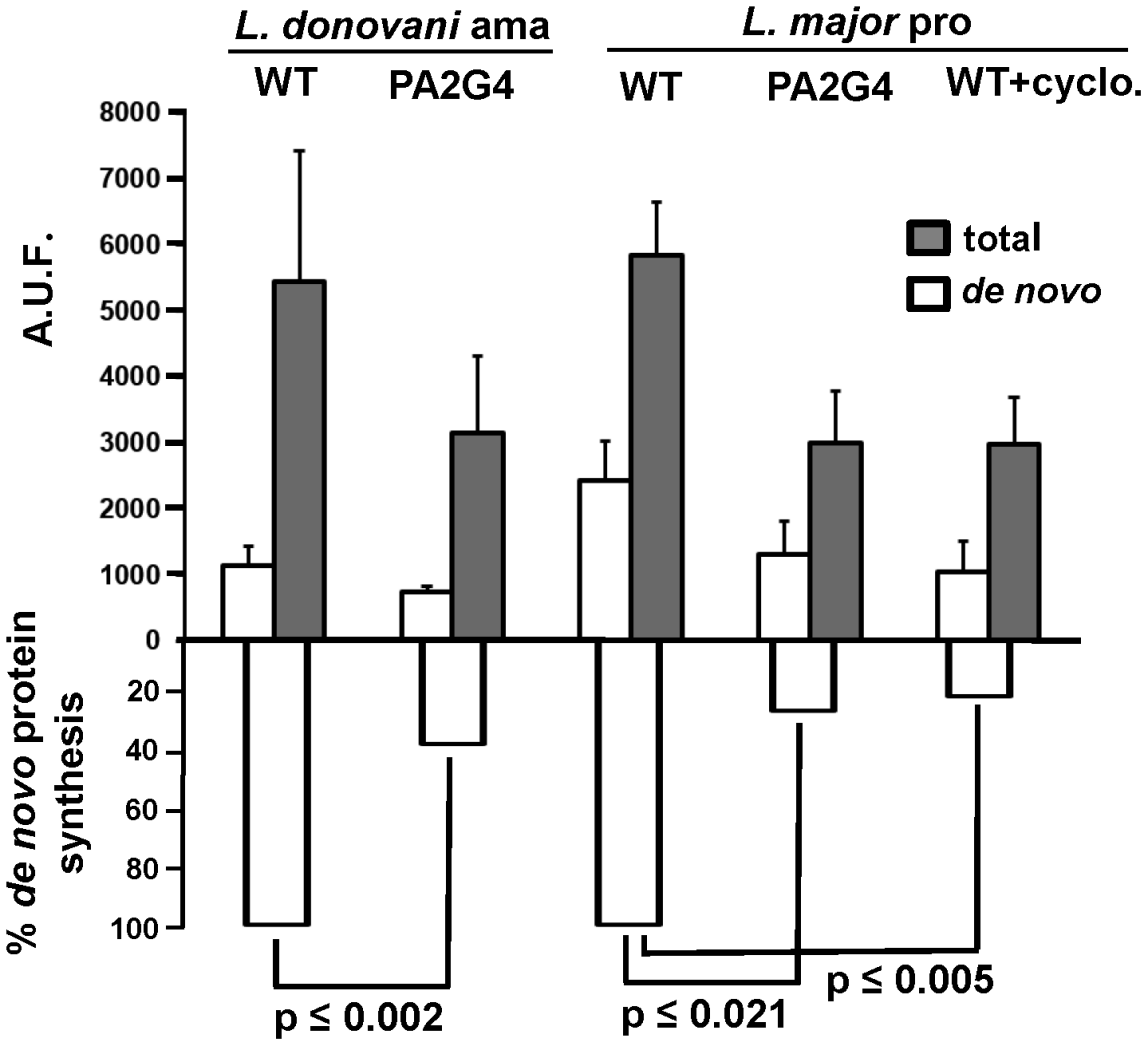
Over-expression of PA2G4 leads to a reduced *de novo* protein synthesis. Metabolic labeling of *de novo* synthetized proteins was conducted using a non-radioactive Click-iT (Life) assay. Mid-log *L. major* WT and GFP-PA2G4 promastigotes (pro) and *L. donovani* WT and GFP-PA2G4 amastigotes (ama) were used in the experiment. Metabolic labeling was performed for 2 h at 27°C in presence of 50 µM azidohomoalanine (AHA). A culture of *L. major* treated for 2 h with 100 µg/mL cycloheximide, an inhibitor of protein biosynthesis, was included as a positive control. Fluorescence signal from TAMRA (Life) was measured to determine *de novo* protein synthesis. Gels were then stained with SYPRO Ruby (Molecular Probes) to quantify the signal of total protein synthesis. Fluorescence intensities are plotted as arbitrary units of fluorescence (AUF). Two independent experiments were performed and one representative triplicate experiment with standard deviation is shown. Percentage of *de novo* synthesis normalized to WT values is shown in the lower panel. Median values were used for non-parametric Mann–Whitney U-test.

The results from last section suggest a defect in protein translation in transgenic parasites, potentially due to the non-physiological accumulation of intermediates of translation initiation and elongation. In order to confirm this phenotype, we measured *de novo* protein synthesis in the transgenic lines. Metabolic labeling of *de novo* synthetized proteins was conducted using a non-radioactive assay. Mid-log *L. major* WT and GFP-PA2G4 promastigotes as well as *L. donovani* WT and GFP-PA2G4 amastigotes were initially incubated in methionine-free medium. Metabolic labeling was performed for 2 h at 27°C in the presence of azidohomoalanine (AHA). A culture of *L. major* treated for 2 h with 100 µg/mL cycloheximide, an inhibitor of protein biosynthesis, was included as a positive control. After labeling, cells were harvested, lysed and subjected to the Click-iT (Life) reaction with TAMRA. Proteins were resolved in polyacrylamide gels and visualized in a Typhoon FLA 9500 imager and fluorescence measured with ImageQuant TL software (GE Healthcare). The gel was then fixed and stained with SYPRO Ruby in order to assess total protein content (Fig. 8). Percentage of *de novo* protein synthesis is shown normalized to the controls *L. donovani* and *L. major* WT parasites (Fig. 8, lower panel). These data suggest than indeed *de novo* protein synthesis is greatly reduced in pro- and amastigotes over-expressing LmaPA2G4. Defects in biosynthesis will likely impact cell proliferation and ultimately may be responsible for the phenotype observed.

## Discussion

LmaPA2G4 is a homolog of the proliferation-associated 2G4 protein [35] also termed Ebp1 [14]. The human counterparts are involved in the regulation of cell growth and differentiation [36]. Human homolog Ebp1 is a target for phosphorylation by PKC *in vitro* and *in vivo*, and its C-terminus has been suggested to harbor the phosphorylation site [37]. Furthermore, serine 363 (S363) of Ebp1 is phosphorylated *in vivo* and the S363A mutation significantly decreased the ability of Ebp1 to repress transcription and abrogated its ability to inhibit cell growth [38]. LmaPA2G4 was isolated through a phospho-enrichment procedure (IMAC), suggesting it may be a phosphoprotein. We are currently characterizing the potential phosphorylated residues in LmaPA2G4 with a combination of IMAC enrichment and 2D LC-MS/MS.

LmaPA2G4 is a metallopeptidase of the M24A family, clan MG (Fig. 2). Bio-informatics analysis and multiple alignment identified members of this family across all trypanosomatids, with a remarkable conservation. The crystal structure of the human PA2G4 has been determined at 1.6 Å resolution [13]. The structure revealed a pita-bread fold conserved in methionine aminopeptidases (MetAPs). In these enzymes, a divalent metal center within the catalytic site is involved in the cleavage of the appropriate substrate. However, the metal ions are not present in PA2G4 [13], and therefore no enzymatic activity can be performed. This group of enzymes are reflected in MEROPS database as non-peptidase members of the M24 family. LmaPA2G4 is well conserved among trypanosomatids, indicating an essential function and selection pressure despite having lost its catalytic site. Further substrate binding studies are necessary to conclusively label LmaPA2G4 as a pseudoenzyme. Inactive enzyme homologs are not simply debris and functional studies, for instance in the iRhom family of rhomboid proteases, have revealed important roles as biological regulators [39]. With the exception of pseudokinases, there is still a lack of functional information on the roles of inactive enzymes [40].

We have confirmed that LmaPA2G4 is an essential gene in *L. major*. Study of some promising candidate genes through loss-of-function is often hindered by lethal mutant phenotypes and our system (Fig. 3A) allows to test whether the guarding episome (pXNG) can be actively and quickly removed in the null mutant (Fig. 3C). Moreover, it indicates that the loss of LmaPA2G4 cannot be compensated by other related genes. Our findings may be applicable to other trypanosomatids, however viability of the LmaPA2G4 null mutant must be carefully examined in *Trypanosoma* and other *Leishmania* spp.

The overexpression of LmaPA2G4 did not impair the ability of *L. major* to differentiate into infective metacyclic promastigotes (Fig. 4D) and *L. donovani* promastigotes were able to fully differentiate into axenic amastigotes within 48 h (Fig. 5B). These data suggest that the attenuated virulence observed in the murine model (Fig. 5A) is likely due to a defect in proliferation. To better understand this phenotype, we performed quantitative proteomics that allowed us to study significant differences in protein expression as a result of LmaPA2G4 overexpression (Fig. 6A and B). 2D DIGE has proven to circumvent the limitation of traditional in-gel proteomics, especially when combined with bottom-up proteomics [41]. The most over-represented −6.1 fold change- spot corresponds to the eukaryotic elongation factor 5A. eIF5A is the only protein that contains the modified amino acid hypusine [42]. Hypusine is formed in eIF5A by post-translational modification of one of the lysyl residues in two consecutive steps through the action of deoxyhypusine synthase (DHS) and deoxyhypusine hydroxylase (DOHH) [43]. The hypusine pathway is conserved in trypanosomatids, and DHS and DOHH have been recently characterized in *T. brucei* [44] and *L. donovani* [45], respectively. In higher eukaryotes, eIF5A has an active role in translation elongation, however its precise requirement in protein synthesis remains elusive [46]. 60S and 40S ribosomal subunits showed a 5.9 and 5.3 fold change respectively in promastigotes over-expressing LmaPA2G4. In higher eukaryotes, translation initiation starts with the disassociation of the 80S ribosomal complex and the binding of eIF6 to the 60S ribosomal subunit and the binding of eIF3 and eIF1A to the 40S ribosomal subunit [47]. The HSP60 family of chaperonines −4.4 fold change in our analysis- are widely present in trypanosomatids and they have a potential role in folding of proteins imported into the mitochondria [48]. It is noteworthy that our proteomic approach does not allow us to confirm any potential interaction of LmaPA2G4 with the key transcription elements discussed above. The EMSA assay suggests that GFP-PA2G4 binds a generic and synthetic double stranded RNA (Fig. 7). It is tempting to speculate that further investigations on the RNA binding domains will allow us to gain insight on the biological relevance of binding on, for instance, translational control. The fact the *de novo* protein synthesis is significantly reduced in the transgenic lines (Fig. 8) and the new insights on transcriptional roles of the human counterpart [49] suggest a potential role of LmaPA2G4 in transcription in *L. major*. Altered transcription in lines over-expressing LmaPA2G4 lines leads to defects in cell growth, including the pathogenic amastigote stage. However, further investigation is required to dissect the molecular mechanisms in which LmaPA2G4 is involved. In conclusion, this work underscores its essential role in the biology of the parasite and opens new venues for potential therapeutic intervention.

## Supporting Information

Figure S1 **In vitro kinase assay.** GFP-PA2G4 and GFPK7 immuno-precipitates were isolated from stationary promastigotes. Phosphotransferase activity was determined by autoradiography. Molecular weight is indicated in kDa. A replica gel was analyzed by western blot using monoclonal GFP antibody. Lane 1: GFP7 incubated with GFP-PA2G4 as a substrate; lane 2: GFPK7 incubated with 3 µg MBP; lane 3: GFP-PA2G4 immuno-complex; lane 4: GFPK7 immuno-complex.
(TIF)

Figure S2 **RT-PCR analysis of PA2G4 transcript levels.** Total RNA was isolated from *L. major* logarithmic (l) and met acyclic (m) promastigotes and from *L. donovani* logarithmic (l) promastigotes and (a) amastigotes. After cDNA synthesis, PCR was performed with specific primers for PA2G4 and GAPDH. Products were resolved in 1% agarose gels, stained with SybrSafe (Life) and scanned on a Typhoon FLA 9500 imager. Band intensities were analyzed with ImageQuant TL (GE Healthcare) and PA2G4 expression was normalized to GAPDH signal. Reactions without RT were used a negative control. Three independent reactions were carried and standard deviations are shown.
(TIF)

Table S1 **Raw data of mass spectrometry identification and Mascot and/or Protein Pilot searches.**
(PDF)
