# Supplementary figures and images for: LmaPA2G4, a Homolog of Human Ebp1, Is an Essential Gene and Inhibits Cell Proliferation in L. major
Source: PLoS Negl Trop Dis. 2014 Jan 9;8(1):e2646. doi: 10.1371/journal.pntd.0002646 (PMC3888471; doi:10.1371/journal.pntd.0002646)

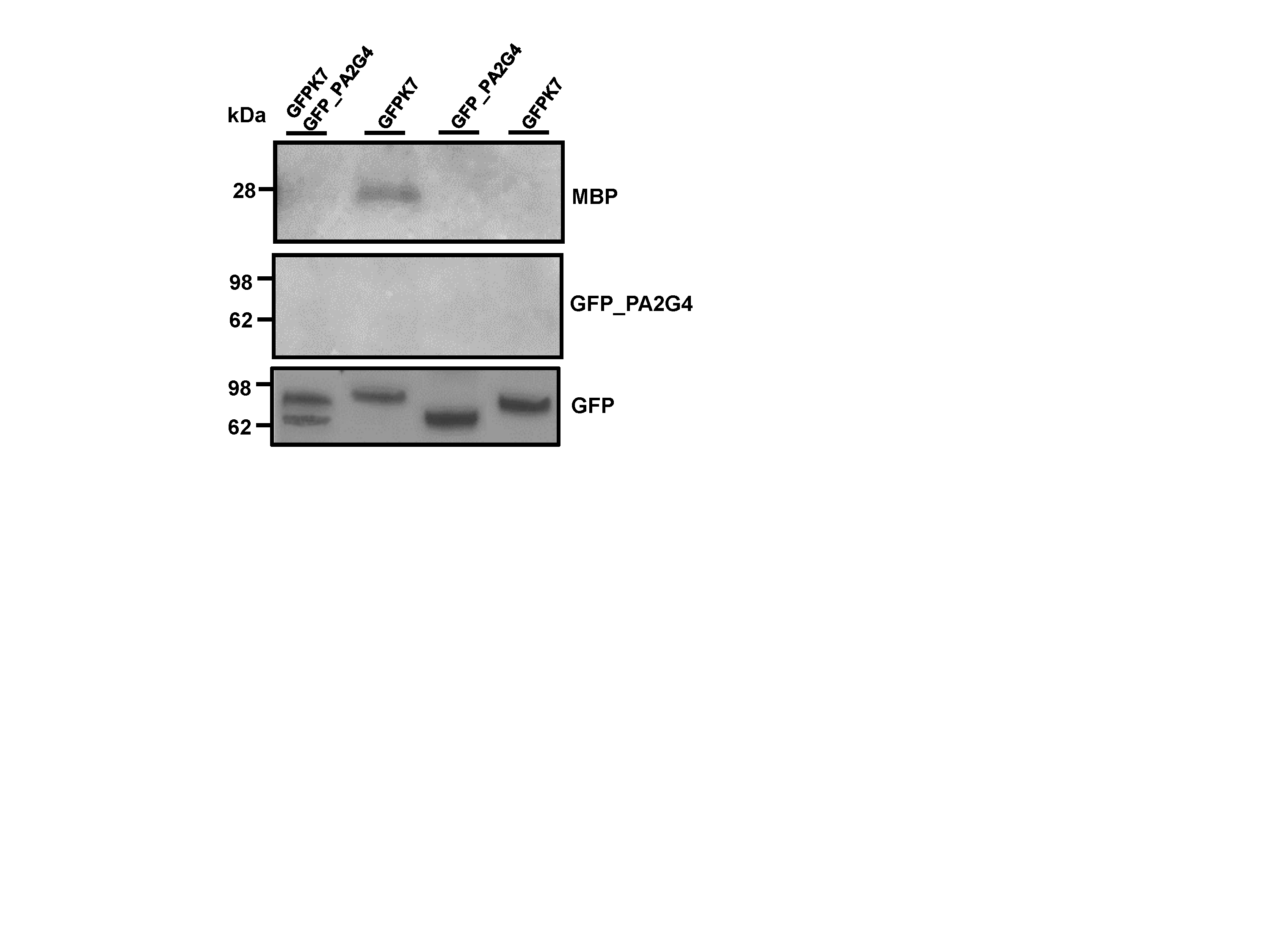

Supplement: Figure S1 — In vitro kinase assay. GFP-PA2G4 and GFPK7 immuno-precipitates were isolated from stationary promastigotes. Phosphotransferase activity was determined by autoradiography. Molecular weight is indicated in kDa. A replica gel was analyzed by western blot using monoclonal GFP antibody. Lane 1: GFP7 incubated with GFP-PA2G4 as a substrate; lane 2: GFPK7 incubated with 3 µg MBP; lane 3: GFP-PA2G4 immuno-complex; lane 4: GFPK7 immuno-complex. (TIF) [file pntd.0002646.s001.tif]

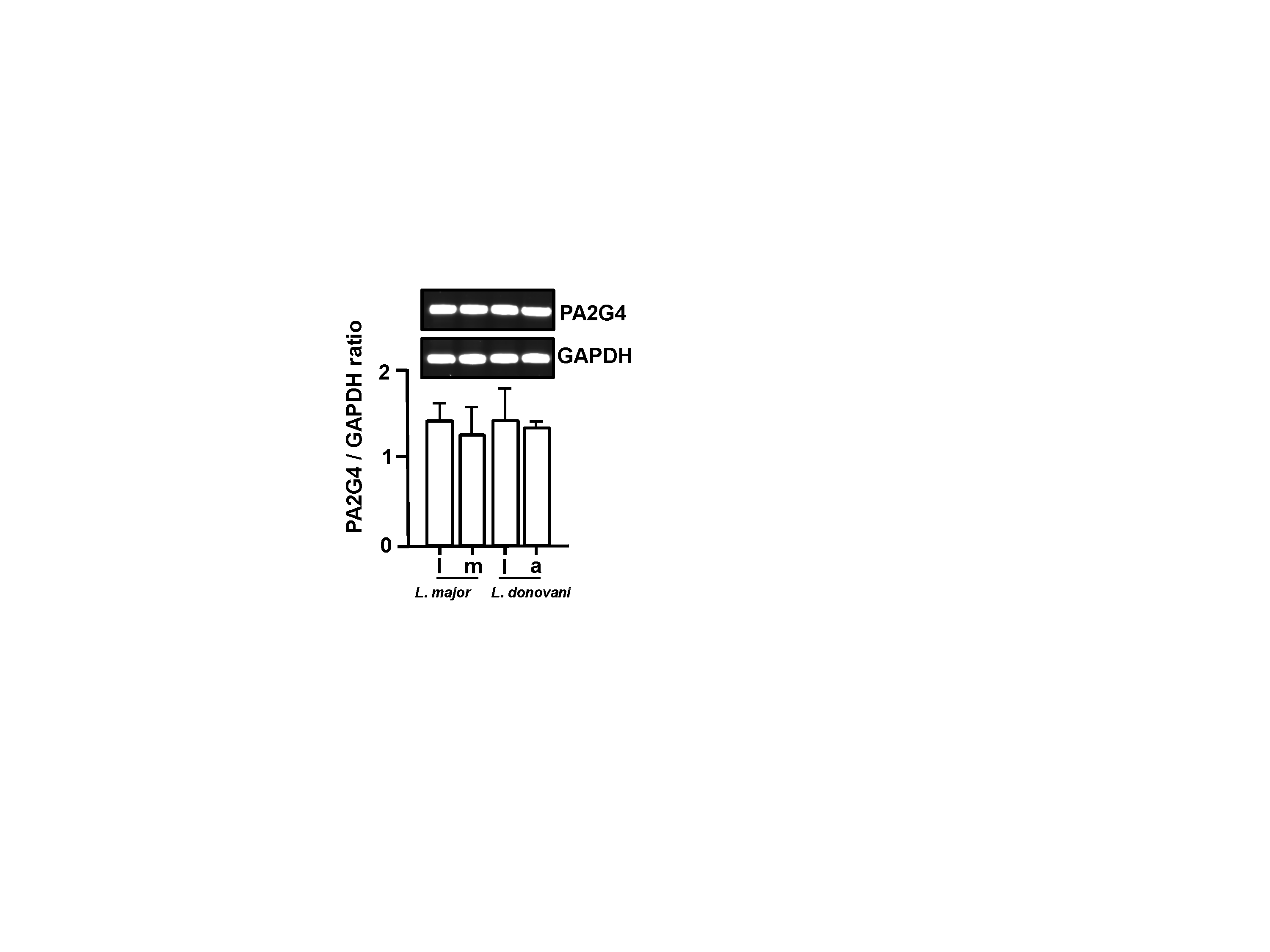

Supplement: Figure S2 — RT-PCR analysis of PA2G4 transcript levels. Total RNA was isolated from L. major logarithmic (l) and met acyclic (m) promastigotes and from L. donovani logarithmic (l) promastigotes and (a) amastigotes. After cDNA synthesis, PCR was performed with specific primers for PA2G4 and GAPDH. Products were resolved in 1% agarose gels, stained with SybrSafe (Life) and scanned on a Typhoon FLA 9500 imager. Band intensities were analyzed with ImageQuant TL (GE Healthcare) and PA2G4 expression was normalized to GAPDH signal. Reactions without RT were used a negative control. Three independent reactions were carried and standard deviations are shown. (TIF) [file pntd.0002646.s002.tif]
