# Supplementary material for: LmaPA2G4, a Homolog of Human Ebp1, Is an Essential Gene and Inhibits Cell Proliferation in L. major
Source: PLoS Negl Trop Dis. 2014 Jan 9;8(1):e2646. doi: 10.1371/journal.pntd.0002646 (PMC3888471; doi:10.1371/journal.pntd.0002646)
Supplement: Table S1 — Raw data of mass spectrometry identification and Mascot and/or Protein Pilot searches. (PDF) [file pntd.0002646.s003.pdf]

Protein Identified	Mass	Mascot Score	Queries Matched
LmjF25.0720 |EIF5A1||eukaryotic initiation factor 5a, putative|Leishmania major|chr 25|||Manual	17915	76	6
LmjF29.2460 |||60S ribosomal protein L13, putative|Leishmania major|chr 29|||Manual	24906	128	3
LmjF11.0960 |||40S ribosomal protein S5, conserved|Leishmania major|chr 11|||Manual	21298	113	4
LmjF36.2030 |||chaperonin Hsp60, mitochondrial precursor|Leishmania major|chr 36|||Manual	59623	281	8
LmjF19.1080 |||hypothetical protein, conserved|Leishmania major|chr 19|||Manual	403741	39	14
